# Loop-Mediated Isothermal Amplification (LAMP) for the Rapid and Sensitive Detection of *Alternaria alternata* (Fr.) Keissl in Apple Alternaria Blotch Disease with *Aapg-1* Encoding the Endopolygalacturonase

**DOI:** 10.3390/pathogens11111221

**Published:** 2022-10-23

**Authors:** Baoyou Liu, Zhiwei Li, Jianfeng Du, Wei Zhang, Xiaozhi Che, Ziran Zhang, Ping Chen, Yingzi Wang, Yang Li, Shaoli Wang, Xinhua Ding

**Affiliations:** 1 State Key Laboratory of Crop Biology, Shandong Provincial Key Laboratory for Biology of Vegetable Diseases and Insect Pests, College of Plant Protection, Shandong Agricultural University, Tai’an 271018, China; 2 Institute of Plant Protection and Resource and Environment, Yantai Academy of Agricultural Sciences, Yantai 265500, China; 3 College of Life Sciences, Yantai University, Yantai 264005, China; 4 Longwangzhuang Sub-District Office of Laiyang City, Yantai 265209, China

**Keywords:** apple Alternaria blotch disease, *Alternaria alternata*, rapid detection, LAMP

## Abstract

Apple Alternaria blotch disease, caused by *Alternaria alternata* (Fr.) Keissl, is one of the most famous leaf diseases. When the disease is prevalent, it causes leaf abscission and influences the formation of flower buds and photosynthesis. Therefore, a simple, rapid, high-specificity and sensitivity method for monitoring infected leaves at early developmental stages is urgently needed, so that the occurrence and expansion of *A. alternata* can be controlled in time. In our research, a rapid, specific and efficient loop-mediated isothermal amplification (LAMP) method was developed to detect *A. alternata* within 60 min. Six primers of LAMP detection can only specifically amplify the *aapg-1* gene in *A. alternata* but not in four other important fungi in apples. The *aapg-1* gene encodes endopolygalacturonase in *A. alternata*, and there are significant differences among different species. Thus, it was applied as the target for LAMP primers. Compared to conventional PCR detection, our LAMP method had the same sensitivity as that of detecting as little as 1 fg of pure genomic DNA of *A. alternata*. When leaves were inoculated with *A. alternata* conidia, LAMP detected 1 × 10^2^ conidia/mL as the minimum concentration. However, the traditional tissue isolation and identification method only isolated *A. alternata* from leaves inoculated with 1 × 10^5^ and 1 × 10^6^ conidia/mL, indicating that the LAMP method was more sensitive than the traditional tissue isolation and identification method for *A. alternata* before symptoms. Further tests also indicated that LAMP detection was more accurate and sensitive than the traditional tissue isolation and identification method for *A. alternata* in leaves with the Alternaria blotch symptom collected from the field. Our results showed that the LAMP-targeting the *aapg-1* gene has the advantages of high sensitivity, specificity and simplicity and can be used for rapid detection and early monitoring of *A. alternata* in the field. LAMP is instructive for us to effectively prevent and control apple Alternaria blotch disease.

## 1. Introduction

Apples (*Malus domestica*) are widely consumed all over the world. As the largest apple cultivation area in the world, China’s total output accounts for more than half of the world’s total apple fruit production [1]. Apple Alternaria blotch disease, also known as brown streak disease, is caused by *A. alternata* [2]. It was first discovered in the United States [3] and is one of the most serious diseases in Asia [4,5]. Apple Alternaria blotch disease causes a loss of up to 50% of apple production [6]. Especially in recent years, the disease has spread rapidly in the main apple growing areas in north China and has become one of the three major leaf diseases in the main apple producing areas in China [7]. When the disease is prevalent, it will cause leaf abscission, resulting in worse growth of the tree, which influences the formation of flower buds and photosynthesis [8]. *A. alternata* can also infect fruits, causing fruit spots after removing the bag and influencing fruit quality [9]. There are significant differences in resistance to apple Alternaria blotch disease among different apple varieties [10]. As the most widely planted variety in China, the ‘Fuji’ apple is infected by *Alternaria alternata* and greatly threatened in terms of the quality and output of apples. Those influences pose significant threats to apple production in China. Thus, in order to stabilize the healthy development of apple cultivation, it is urgent to prevent the occurrence and spread of *A. alternata*.

Apple Alternaria blotch disease is an airborne disease, and mainly infects the leaves, especially the 20-day-old leaves. Sometimes it also infects fruits and shoots. By producing AM toxin, *A. alternata* acts on apple leaves, affecting the normal growth of leaves and then making them susceptible to the disease [11,12,13,14]. Brown or black lesions with a diameter of 2~5 mm will appear after infection, which causes leaves to dry and fall over, seriously affecting yield [15]. The infection sources of *A. alternata* are very extensive, mainly including mycelia in deciduous leaves and dead branches. *A. alternata* produces spores in May of the following year and spreads with the wind and rain. With the growth of shoots, the disease peaks in July and August [16,17]. *A. alternata* can also infect the fruit after taking off the outer bags in October, which causes red spots on the peel and affects the quality of the fruit [9]. However, the prevention and control of apple Alternaria blotch disease is mainly based on chemical control [18]. Many kinds of chemical agents are used, but there is a lack of evidence and related studies to determine the accurate application time. The continuous use of fungicides will lead to the emergence of many resistant strains and increase the difficulty of disease prevention and control. In order to reduce the use of fungicide and improve the chemical control effect, it is necessary to monitor *A. alternata* before the symptoms appear.

Several methods, including *A. alternata* isolation, hyphae and spore morphology scanning [19], and polymerase chain reaction (PCR) detection [20], are common and practical for the diagnosis of apple Alternaria blotch disease in the laboratory. However, these traditional identification methods are unsuitable for application in the field, as they require specialized technology and equipment, such as microscopes and PCR thermocycle instruments. Moreover, isolating and culturing fungi is time-consuming. Although PCR detection is shorter than traditional isolation methods, it requires expensive equipment, such as PCR instruments and gel imagers. Based on these shortcomings, loop-mediated isothermal amplification (LAMP) was established in our research for the detection of *A. alternata*.

LAMP was first invented and applied in 2000, which is a rapid, specific and efficient method for the amplification of DNA sequences at a stable temperature [21]. LAMP detection is less sensitive to some inhibitors, such as metal ions and protease, than traditional PCR and has been applied for detection of some plant fungi, including *Didymella bryoniae* from cucurbit seeds [22] and *Colletotrichum truncatum* from soybeans [23]. Further development of LAMP involves the combination of this technology with other molecular methods, such as reverse transcription and multiplex amplification, for detection of infectious diseases caused by microorganisms in humans, livestock and plants [24]. LAMP detection applies a set of four to six primers and *Bst* DNA polymerase with strand displacement activity to amplify target DNA sequences with high specificity and no DNA denaturation stage at a stable temperature [25]. The product and by-product (magnesium pyrophosphate) of the LAMP reaction can be detected by visual assessment of turbidity or a color change with the addition of color-changing reagents, such as SYBR-Green I and HNB [26]. LAMP products can also be visualized on agarose gel as a banding pattern [27]. In general, without specialized technology and equipment, LAMP assays can amplify DNA with high specificity and efficiency. Moreover, testing with a water bath or heating block and the color change of products makes LAMP detection suitable in the field or in limited-resource settings [28].

In this research, we established a rapid, specific and efficient method for the detection of *A. alternata* in apple leaves based on the *aapg-1* gene, which is unique to *A. alternata* and encodes endopolygalacturonase, playing an important role in plant cell wall degradation when fungi infect plants [29]. Finally, the early and rapid LAMP detection can be used to monitor *A. alternata* and control its development over time.

## 2. Results

### 2.1. LAMP Primers

**Figure 1:**
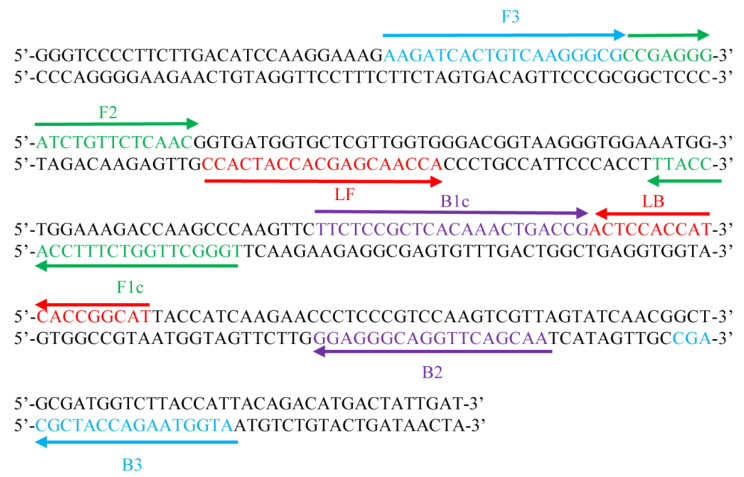
Partial sequence of endopolygalacturonase (*aapg-1*) of *Alternaria alternata* and the location of the LAMP primers. Arrows indicate the direction of extension. Six specific primers targeting eight conserved regions of *aapg-1* include two outer (F3 and B3) and two inner [FIP (Forward Inner Primer, F1c and F2) and BIP (Backward Inner Primer, B1c and B2)] primers and loop primers (LF and LB) were designed.

The LAMP primers were designed with the target sequence (250 bp) of the *aapg-1* gene encoding endopolygalacturonase (AB047682.1) in *A. alternate* (Figure 1, Table 1). The target sequence was selected from a region of high homology by comparison with several sequences belonging to *Alternaria*. The primers exhibiting high specificity and sensitivity did not show similarities to any other fungi sequences. For LAMP primers, ΔG values of 3′ ends of F3/B3 primer, F2/B2 primer and LF/LB primer, and 5′ ends of F1c and B1c primer were determined, and the values were −7.36, −7.42, −4.51, −6.14, −4.90, −6.57, −6.59 and −4.71 Kcal/mole, respectively. All ΔG values were less than −4 Kcal/mol.

### 2.2. Specificity and Sensitivity of LAMP Detection

**Figure 2:**
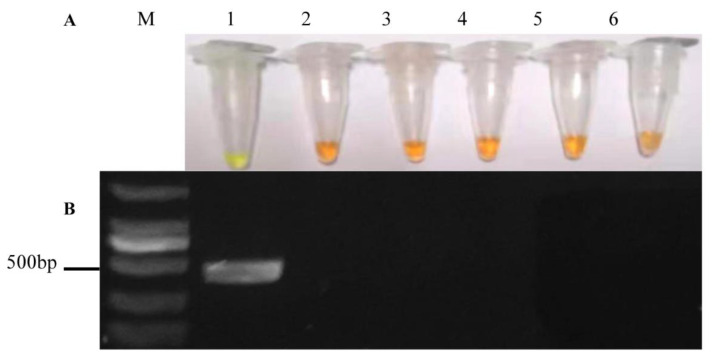
Specificity assay of LAMP and PCR primers in amplifying of the *aapg-1* gene in *Alternaria alternata*. (**A**) Visual evaluation of LAMP products’ color change based on SYBR Green I; (**B**) Banding pattern on agarose gel electrophoresis of PCR products. M, Marker DL 2000; 1, *A. alternata*; 2, *Botryosphaeria dothidea*; 3, *Glomerella cingulata*; 4, *Diplocarpon mali*; 5, *Trichothecium roseum*; 6, nuclease-free water.

The specificity of LAMP primers was tested with mycelial DNA of *A. alternata* and four important pathogenic fungi of apples, i.e., *Botryosphaeria dothidea*, *Glomerella cingulata*, *Diplocarpon mali*, and *Trichothecium roseum*. In order to verify the results of LAMP detection, we amplified *A. alternata* and the other four non-target pathogenic fungi with PCR primers (Table 1). The results of LAMP detection can be visualized via color change from orange to green by adding SYBR Green I. The detection of *A. alternata* was positive in each repeat, and the color of the reaction solution changed markedly from orange to green, while the other fungi remained an orange color. The nuclease-free water templates showed no color change in any validation test (Figure 2A). Moreover, a 440 bp product in gel electrophoresis of the PCR amplification indicated the same results as the color change of LAMP detection (Figure 2B). Consequently, the newly established LAMP detection using six primers (Table 1) showed high specificity in the detection of *A. alternata*.

After it was determined that the primers were specific for *A. alternata*, the lowest detection limit was carried out using 10-fold serial dilutions of pure *A. alternata* mycelial DNA (1 ng to 1 ag).

**Figure 3:**
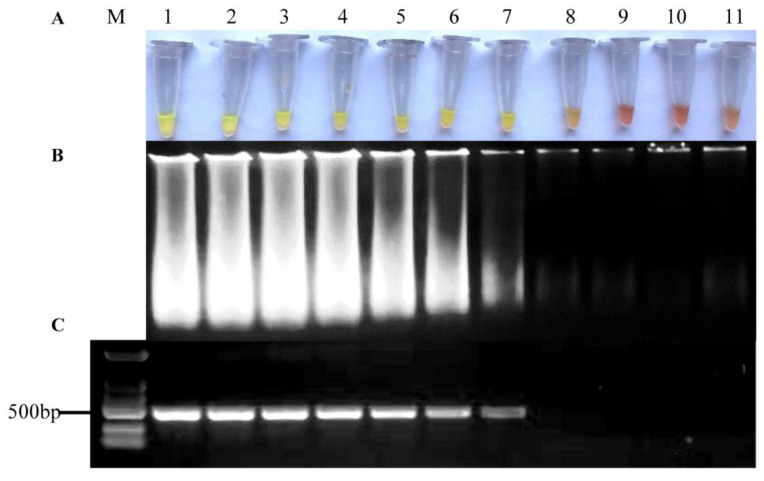
The results of LAMP detection with different concentrations of the DNA template. (**A**) Visualization of color change of the LAMP products based on SYBR Green I; (**B**) Analysis of the LAMP products based on gel electrophoresis; (**C**) Analysis of the PCR products based on gel electrophoresis. M, DL2000 DNA marker; 1, 1 ng/μL; 2, 100 pg/μL; 3, 10 pg/μL; 4, 1 pg/μL; 5, 100 fg/μL; 6, 10 fg/μL; 7, 1 fg/μL; 8, 100 ag/μL; 9, 10 ag/μL; 10, 1 ag/μL; 11, nuclease-free water.

The lowest detection limit for *A. alternata* was 1 fg of pure *A. alternata* mycelial DNA as a template within 60 min, along with color change by adding SYBR Green I (Figure 3A) and diffuse type bands on gel electrophoresis (Figure 3B). As a comparison, conventional PCR detection using primers aapg-1-F/aapg-1-R (Table 1) exhibited the same lowest detection limit as the LAMP assay (Figure 3C).

### 2.3. LAMP Detection of the Minimum Pathogenic Concentration of A. alternata Conidia in Apple Leaves

**Figure 4:**
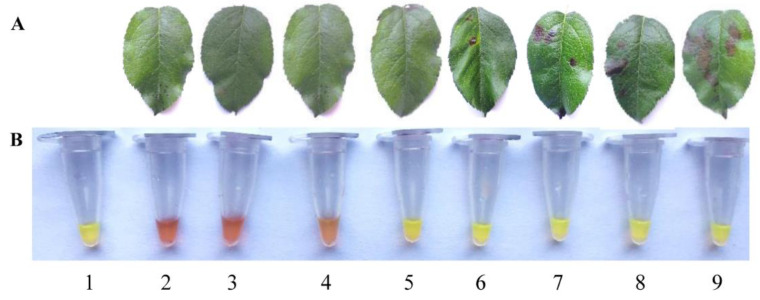
The detection of minimum pathogenic concentration of *Alternaria alternata* conidia by LAMP. (**A**) Symptoms of apple leaf samples with different treatments; (**B**) Detection of *A. alternata* conidia by LAMP. 1, DNA from *A. alternata*; 2, DNA from inoculated apple leaf samples treated with 3 μg/mL of tebuconazole; 3, DNA from inoculated apple leaf samples treated with nuclease-free water; 4–9, DNA of apple leaf samples incubated with 1 × 10^1^, 1 × 10^2^, 1 × 10^3^, 1 × 10^4^, 1 × 10^5^ and 1 × 10^6^ *A. alternata* conidia/mL.

Necrotic spot symptoms were observed after 3 days of incubation in the leaves incubated with 1 × 10^3^ conidia/mL to 1 × 10^6^ conidia/mL inoculum suspension. Negative controls incubated with tebuconazole [30] and nuclease-free water were still healthy (Figure 4A). Then, we succeeded in isolating *A. alternata* only from leaves incubated with 1 × 10^3^ conidia/mL to 1 × 10^6^ conidia/mL inoculum suspension by the traditional isolation method with a frequency of 60.9%, 62.8%, 69.5% and 75.3%. However, the LAMP detection of leaves incubated with inoculum suspension of *A. alternata* at a concentration of 1 × 10^2^ conidia/mL to 1 × 10^6^ conidia/mL and the positive control of *A. alternata* DNA were all positive with a frequency of 100% (Figure 4B). Those results suggested that LAMP detection was more accurate and showed higher sensitivity than the traditional isolation method.

### 2.4. LAMP, PCR and Traditional Isolation Method to Detect A. alternata in Leaf Samples Collected from the Field

**Figure 5:**
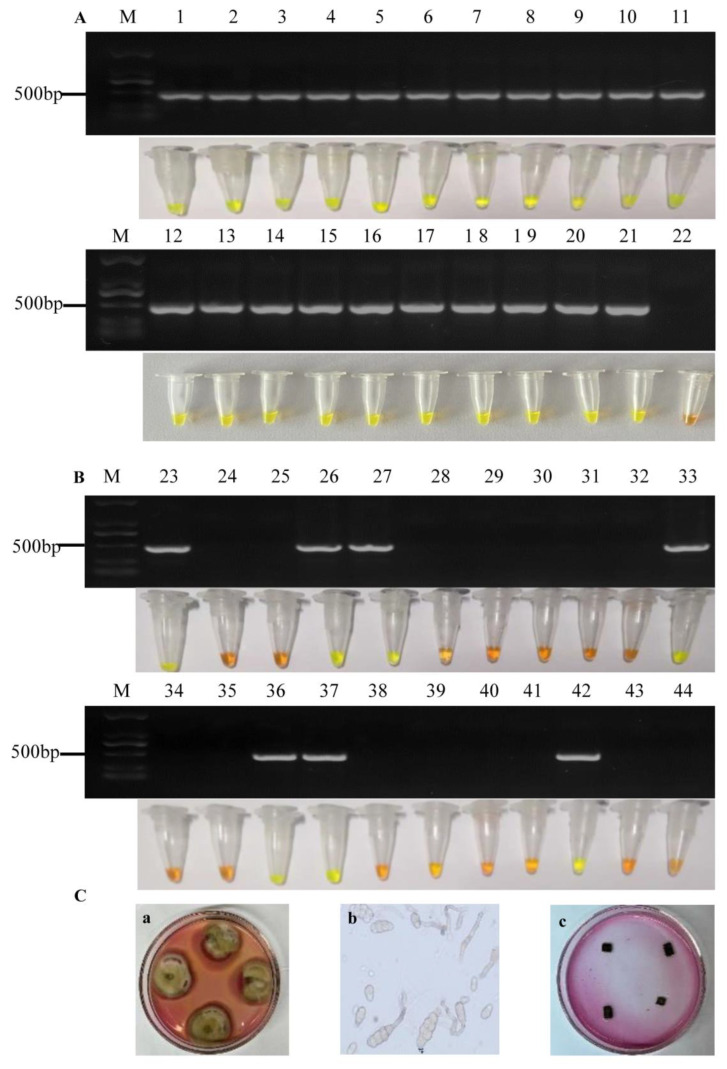
PCR and LAMP Detection of *Alternaria alternata* in apple leaf samples with Alternaria blotch symptoms and healthy apple leaf samples collected from the field. (**A**) PCR and LAMP detection of *A. alternata* in apple leaf samples with Alternaria blotch symptom; (**B**) PCR and LAMP detection of *A. alternata* in healthy apple leaf samples. M, DL2000 DNA marker; 1, 23, DNA extracted from *A. alternata*; 2–21, DNA extracted from apple leaf samples with Alternaria blotch symptom; 24–43, DNA extracted from healthy apple leaf samples; 22, 44, nuclease-free water; (**C**) Colony and spore photographs of *A. alternata* identified by traditional isolation method. (**a**): colony photograph of *A. alternata* strain from sample 16 identified by the traditional isolation method; (**b**): spore photograph of *A. alternate* strain from sample 16 identified by the traditional isolation method. (**c**): plate without any colonies from sample 39 identified by the traditional isolation method.

We collected 20 suspected apple Alternaria blotch samples from the field. LAMP, PCR and the traditional isolation method were applied to detect *A. alternata* in those leaves. As shown in Figure 5A, 20 DNA templates numbered 2–21 from suspected apple Alternaria blotch samples with necrotic spot symptoms and the positive control DNA templates numbered 1 from mycelium of *A. alternata* were all detected with 440 bp products by PCR and displayed a green color in the LAMP assay. For the traditional tissue isolation and identification method, *A. alternata* was isolated from 16 suspected apple Alternaria blotch samples by observing colony and spore morphology. Colony photographs of some *A. alternata* strains identified by the traditional isolation method were shown in Figure 5C. Those results showed that there is the same detection rate between LAMP and PCR detection for suspected apple Alternaria blotch samples, both of which are higher than the traditional isolation method.

Furthermore, in order to carry out the early prevention and control of *A. alternata* before symptoms appear, we collected 20 healthy leaf samples from the field. LAMP, PCR and the traditional isolation method were applied to detect *A. alternata* in those healthy leaves. As shown in Figure 5B, DNA templates from healthy leaves numbered 26, 27, 33, 36, 37 and 42, and the positive control DNA templates numbered 23 from *A. alternata* were all detected with 440 bp products by PCR and displayed green color by LAMP assay. For the traditional tissue isolation and identification method, *A. alternata* was isolated only from the leaves numbered 26 and 27. Although the leaves did not have any symptoms, *Alternaria alternata* was still detected, which also confirmed the presence of *Alternaria alternata* in the leaves.

**Figure 6:**
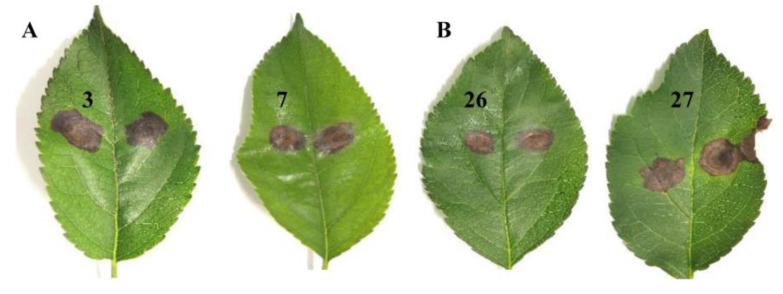
Koch’s rule was used to verify *Alternaria alternata* in apple leaf samples. (**A**) *A. alternata* strains isolated from samples 3 and 7 of apple Alternaria blotch leaves successfully caused leaf spot symptoms; (**B**) *A. alternata* strains isolated from samples 26 and 27 of healthy leaves successfully caused leaf spot symptoms.

According to Koch’s rule, we further cultured all strains of *A. alternata* isolated by the traditional isolation method and re-inoculated apple leaves. The results showed that all strains obtained by the traditional method successfully caused leaf spot symptoms like in Apple Alternaria blotch disease (Figure 6). Those results showed that, compared with the traditional tissue isolation and identification method, our LAMP detection was more accurate and sensitive when used for early prevention and control of *A. alternata* in the field.

## 3. Discussion

Apple Alternaria blotch disease is one of the most important early deciduous diseases in apples, which can cause leaf shedding and influence flower bud formation [31]. It has become one of the main leaf diseases affecting apple production [32]. Apple Alternaria blotch disease and other early leaf litter diseases such as brown spot and anthrax leaf blight have similar early symptoms, so it is difficult to distinguish them by sight [31]. However, the operation of the traditional tissue separation and identification methods is complex and time-consuming. At the same time, due to the different control agents for these early deciduous diseases, the wrong diagnosis will lead to the failure of disease control [33]. Therefore, it is very important to establish a rapid, sensitive and simple detection method for the early detection of *A. alternata* and control of apple Alternaria blotch disease.

Most LAMP detections use the *ITS* gene, which is highly conserved in different fungi, to design primers [34,35]. *ITS* genes have less intraspecific variability and might hinder the development of specific primers for different species. The *aapg-1* gene encodes endopolygalacturonase, which plays an important role in the process of fungal infection in plants [36]. We designed six specific LAMP primers according to the conserved sequence of the *aapg-1* gene and successfully performed LAMP detection for *A. alternata*. These results indicate that the *aapg-1* gene is a highly-specific target gene suitable for its LAMP detection.

LAMP detection can be used to specifically detect *A. alternata* from several important pathogens of apples (Figure 2). Although the detection limit of 1 fg of *A. alternata* DNA of LAMP detection was consistent with that of conventional PCR, LMAP detection is simpler and does not require expensive and complex instruments for reaction and product detection [37]. In addition, the detection limit of LAMP in this study is close to that of *Talaromyces favus* [38]. This result is higher than in other reports, such as the LAMP assay that detected *Soybean mosaic virus* with the lowest limit of 10^−4^ ng/μL [39]. Since LAMP has such high sensitivity to mycelial DNA, we further carried out detection of its minimum pathogenic concentration of spores. The results confirmed that the lowest concentration of spores detected by LAMP was 10^2^ conidia/mL (Figure 4B). The lowest detected concentration of *A. alternata* spores in our study is higher than that of *Colletotrichum gloeosporioides* spores, which is 100 conidia/μL [40]. More importantly, we detected *A. alternata* by LAMP before symptom appearance, so as to monitor *A. alternata* and prevent the disease spread by fungicide in time. This inference is also consistent with the results of successfully detecting *Soybean mosaic virus* before the symptoms [39]. This conclusion was consistent with no disease symptoms on leaves sprayed with tebuconazole and *A. alternata* spores.

Since the early symptoms of *A. alternata* are mild, it is difficult to determine the species of the spots by sight [31]. Moreover, the traditional tissue isolation and identification method is time-consuming and has low accuracy, which leads to the difficulty of early identification of *A. alternata* in the field. The detection rates of LAMP and PCR in this study were higher than those of traditional tissue isolation and identification methods (Figure 5), which was consistent with the detection of pathogenic fungi in most reports, such as the LAMP assay that detected *Didymella bryoniae* in cucurbit seeds with higher accuracy than that of real-time PCR [22]. Sixty-one diseased soybean samples of *Colletotrichum truncatum* were successfully detected from 154 suspected samples using the LAMP assay, but only 29 samples were identified by traditional isolation and culture [23]. The detection rates for *Phytophthora capsici* by LAMP, PCR and the traditional isolation method were 55.4%, 57.8% and 25.3% [41]. The detection rate of *Botryosphaeria dothidea* by the LAMP method was 68%, while the rate of the traditional isolation method was only 24% [42].

Although LAMP detection possesses the above advantages, it is susceptible to atmospheric aerosol contamination and has the risk of false positions [43]. Therefore, this study adopted the method of non-opening detection; that is, SYBR Green I dye was added to the PCR tube cap before the reaction, and the color change was displayed by mixing SYBR Green I dye and the reaction product [44]. However, before LAMP detection, it is still necessary to strictly divide the districts of DNA extraction and LAMP detection. The sterilization of reaction utensils should be strictly sterilized to prevent contamination. Moreover, to avoid the influence of subjective observation, we placed the tubes on a black table or white paper so that the color change would be more obvious. In conclusion, LAMP targeting the *aapg-1* gene has the advantages of high sensitivity, specificity and simplicity, which can be used for rapid detection and early monitoring of *A. alternata* in the field.

## 4. Materials and Methods

### 4.1. Fungal Isolates, Culture Conditions and DNA Extraction

We separated *A. alternata* and four important apple pathogenic fungi, including *Botryosphaeria dothidea*, *Glomerella cingulata*, *Diplocarpon mali* and *Trichothecium roseumisolates*, from diseased apple samples in Yantai, China.

Those fungi were identified using morphological and molecular methods by sequencing ITS sequences. Before all the experiments, all the fungi were transferred to PDA plates and were cultured for 5 d at 25 °C in darkness. Genomic DNA was extracted from each sample using a rapid fungal genomic DNA isolation kit (Sangon Biotech, Shanghai, China) according to the manufacturer’s instructions. The quality of the DNA was checked in agarose gels (1.2%) and the quantity was determined in a spectrophotometer (NanoDrop Technologies, Wilmington, DE, USA). The results of quality and quantity detection of the genomic DNA are shown in Figure S1.

### 4.2. LAMP Primers Design and Screen

The *aapg-1* gene is conserved in *A. alternata*, encodes endopolygalacturonase, and plays an important role in plant cell wall degradation during fungal infection. Therefore, the *aapg-1* gene, as an important pathogenic factor, was chosen to be the specific target for the design of LAMP primers for the detection of *A. alternata*. The LAMP primers, comprising two outer (F3 and B3), two inner (FIP and BIP) primers and two loop primers (LF and LB), were designed using the Primer Explorer V5 software program (http://primerexplorer.jp/lampv5e/index.html, accessed on 5 March 2021) based on the *A. alternata aapg-1* sequence (AB047682.1). The selection of best primers was based on ΔG values of less than or equal to −4 Kcal/mol at the 3′ end of F3/B3, F2/B2, LF/LB and 5′ end of F1c and B1c, which were all synthesized by Sangon Biotech (Shanghai China) Co., Ltd.

### 4.3. LAMP and PCR Reaction Mixtures and Conditions

LAMP detection was performed using the above primers shown in Figure 1 and Table 1. Each reaction contained the target DNA sample 1 μL, 2 × LAMP PCR Master Mix 10 μL, 8 U/μL *Bst* DNA Polymerase 0.5 μL, 10 mM FIP/BIP 2 μL, 10 mM F3/B3 0.5 μL, 10 mM LF/LB 1 μL, adding ddH_2_O to 20 μL. Then 1 μL SYBR Green I was added to the cover of the tube. The reaction mixtures were incubated in a heated block at 65 °C for 60 min followed by incubation at 80 °C for 10 min to terminate the reactions. After the reaction, the results were examined via visual color changes of SYBR Green I (from orange to green) and confirmed by 1.2% agarose gel electrophoresis. PCR reactions were performed using the above primers shown in Table 1. Each reaction contained the target DNA sample 1 μL, 8 U/μL r*Taq* DNA Polymerase 0.2 μL, 10 mM aapg-1-F/aapg-1-R 0.5 μL, 2.5 mM dNTPs 2 µL, 25 mM MgSO_4_ 0.2 µL, 10 × PCR buffer 2 μL, and water to make up the rest to 25 µL adding ddH_2_O to 25 μL. The PCR program was set as: 94 °C for 3 min, followed by 30 cycles of 94 °C for 30 s/cycle, 55 °C for 30 s, and 72 °C for 10 min. The reaction results were examined via 1.2% agarose gel electrophoresis.

### 4.4. Assay of Specificity and Sensitivity of LAMP and PCR Detection

The specificity was determined by the LAMP and PCR reaction mixtures with the conditions mentioned above with DNA extracted from *A. alternata* and four common and important pathogenic fungi of apple. Genomic DNA of *A. alternata* (AB047682.1) was used to determine the sensitivity of the LAMP and PCR detection. The LAMP and PCR detection limits were defined by the smallest amount of DNA detected in each replicate.

Ten-fold serial dilutions of genomic DNA ranging from 1 ng/μL to 1 ag/μL were used as templates for sensitivity detection. Dilution series were prepared with ddH_2_O. LAMP and PCR detections were performed using the same conditions mentioned above. In order to obtain consistent results, each LAMP and PCR reaction was repeated in triplicate. Negative controls contained nuclease-free water in place of genomic DNA. All reactions were performed three times.

### 4.5. Inoculation of Apple Leaves with A. alternata Conidia

Conidia of *A. alternata* were collected from 7-day-old cultures on PDA medium containing Bengal red and suspended in sterile distilled water. The conidial suspensions were determined using a hemocytometer and adjusted to concentrations of 1 × 10, 1 × 10^2^, 1 × 10^3^, 1 × 10^4^, 1 × 10^5^ and 1 × 10^6^ conidial/mL. Inoculation of apple leaves with conidial suspensions refers to previously reported methods [10,15,45] with some modifications. Leaves, which were 20 to 25 days old, were rinsed with running water and the leaf surface was sterilized with 70% alcohol. Then, the leaves were rinsed with sterile water for 3 times and dried the surface water with sterile filter paper. The leaves were immersed in conidial suspensions of different concentrations for 5 min. Negative controls were ddH_2_O in place of conidial suspension. The petiole was covered with absorbent cotton soaked in water and the inoculated leaves were placed in a light incubator with a 12-h photoperiod and a daytime temperature of 28 °C and 25 °C at night (70–80% RH). Each replicate contained 20 leaves [46].

### 4.6. Detection of A. alternata from Artificially Infested Leaves

To assess the detection result by LAMP, the traditional isolation method and the PCR methods, infected and control leaves were both harvested at the same time after inoculation. At 3 days after inoculation, four pieces about 0.5 cm × 0.5 cm were taken from each leaf, immersed in 4% sodium hypochlorite for 4 min, 70% alcohol for 10 s, and rinsed three times in sterile water. Then, those pieces were transferred to PDA medium containing Bengal red and were incubated for 5 d at 25 °C in darkness for traditional isolation and morphological identification. For LAMP detection, four pieces of about 0.5 cm × 0.5 cm were taken from each leaf for DNA extraction with the method mentioned above. Purified DNA from the mycelium of *A. alternata* was used as a positive control, while DNA from non-inoculated leaves was used as a negative control.

### 4.7. Detection of A. alternata from Leaves Collected in Fields

To further confirm the efficiency of LAMP detection for *A. alternata* from leaves, 20 naturally infected leaves with necrotic spot symptoms and 20 healthy leaves without necrotic spot symptoms were collected from the fields in Yantai, Shandong Province. Twelve pieces of each leaf were cut for testing; four pieces for LAMP detection, four pieces for PCR detection, and four pieces for traditionally isolated detection as described above.

### 4.8. Verification of A. alternata Isolated from Leaf Samples by Koch’s Rule

Healthy leaves that were 25 days old and of the same size were picked and washed with sterile water and quickly air dried. Wounds were made by needling the back of leaves with sterilized insect needles. *A. alternata* cultured for 5 days was inoculated and the side with hypha was attached to the wound. Five leaves were inoculated with each strain and a blank cake. The inoculated leaves were placed in a light incubator with a 12-h photoperiod at 28 °C during the daytime and 25 °C at night (70–80% RH).

## Figures and Tables

**Table 1:** Primers used for LAMP and PCR specific detection of *Alternaria alternata*.

Primer Name	Purpose	Sequence (5′-3′)
F3	LAMP detection	AAGATCACTGTCAAGGGCG
B3	LAMP detection	ATGGTAAGACCATCGCAGC
FIP (F1c-F2) *	LAMP detection	TGGGCTTGGTCTTTCCACCATTCCGAGGGATCTGTTCTCAAC
BIP (B1c-B2) *	LAMP detection	TTCTCCGCTCACAAACTGACCGAACGACTTGGACGGGAGG
LF	LAMP detection	ACCAACGAGCACCATCACC
LB	LAMP detection	ACTCCACCATCACCGGCAT
aapg-1-F	PCR detection	CGTCCCTTCAGGCACAACTT
aapg-1-R	PCR detection	AAACCTTAGCGCCATCAATG

* FIP is a hybrid primer composed of the F1c and the F2 sequences, BIP is a hybrid primer composed of the B1c and the B2 sequences.

## Data Availability

All data supporting the findings of this study are available within the paper.

## Supplementary Materials

The following supporting information can be downloaded at: https://www.mdpi.com/article/10.3390/pathogens11111221/s1. Figure S1: The quality and quantity detection of the genomic DNA.
