# Supplementary material for: Loop-Mediated Isothermal Amplification (LAMP) for the Rapid and Sensitive Detection of Alternaria alternata (Fr.) Keissl in Apple Alternaria Blotch Disease with Aapg-1 Encoding the Endopolygalacturonase
Source: Pathogens. 2022 Oct 23;11(11):1221. doi: 10.3390/pathogens11111221 (PMC9697310; doi:10.3390/pathogens11111221)
Supplement: Supplementary file 1 [file pathogens-11-01221-s001.zip › pathogens-1964227-supplementary.pdf]

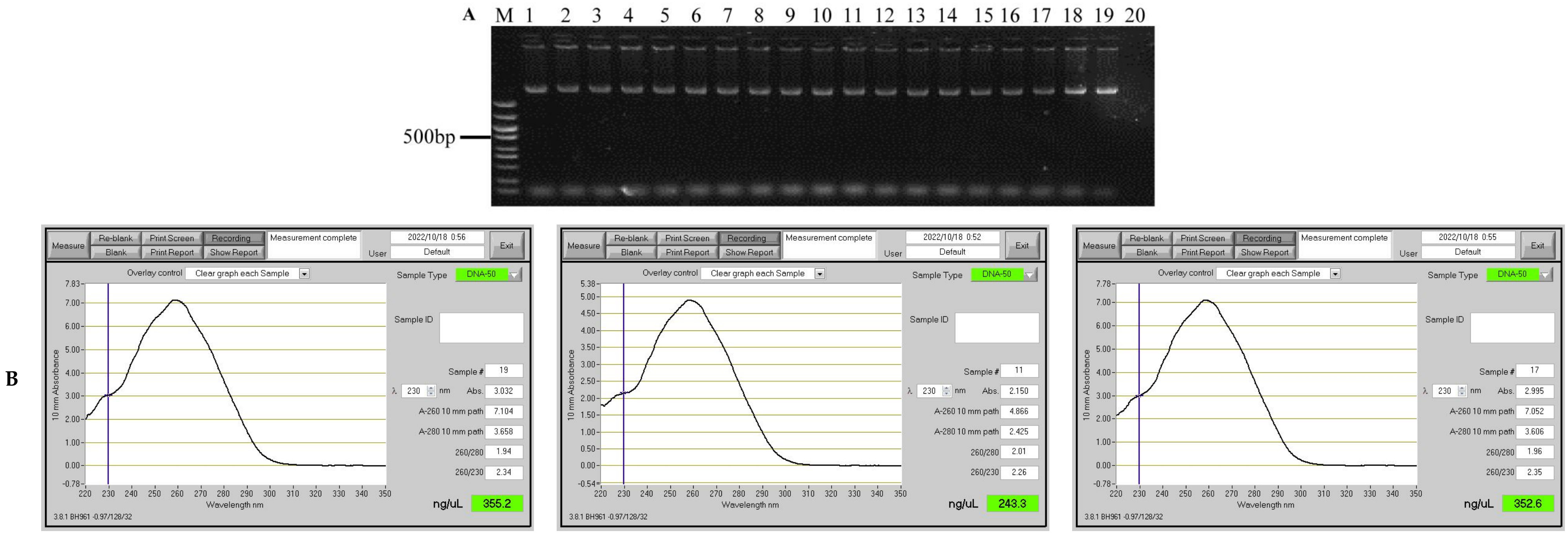

Figure S1: The quality and quantity detection of the genomic DNA. (A) The quality detection of the genomic DNA from samples 1-19 in agarose gels; M, DL2000 DNA marker; 20, nuclease-free water; (B) The quantity detection of the genomic DNA from samples 3, 5 ,10 in a spectrophotometer.
